# Critical shoulder angle and failure of conservative treatment in patients with atraumatic full thickness rotator cuff tears

**DOI:** 10.1186/s12891-022-05519-y

**Published:** 2022-06-10

**Authors:** Arturo Meissner-Haecker, Julio Contreras, Alfonso Valenzuela, Byron Delgado, Angelinni Taglioni, Rodrigo De Marinis, Claudio Calvo, Francisco Soza, Rodrigo Liendo

**Affiliations:** 1 Department of Orthopaedic Surgery, School of Medicine, Pontificia Universidad Catolica de Chile, Santiago, Chile; 2 Instituto Traumatológico, Santiago, Chile; 3 Hospital Van Buren, Valparaiso, Chile; 4 Hospital Dr. Sotero del Rio, Santiago, Chile; 5 Hospital La Florida, Santiago, Chile

**Keywords:** Rotator cuff, Atraumatic full-thickness rotator cuff tear, Shoulder impingement syndrome, Critical shoulder angle, Conservative treatment

## Abstract

**Background:**

Atraumatic full thickness rotator cuff tears (AFTRCT) are common lesions whose incidence increases with age. Physical therapy is an effective conservative treatment in these patients with a reported success rate near 85% within 12 weeks of treatment. The critical shoulder angle (CSA) is a radiographic metric that relates the glenoid inclination with the lateral extension of the acromion in the coronal plane. A larger CSA has been associated with higher incidence of AFTRCT and a higher re-tear rate after surgical treatment. However, no study has yet described an association between a larger CSA and failure of conservatory treatment in ARCT. The main objective of this study is to determine whether there is an association between CSA and failure of physical therapy in patients with AFTRCT.

**Methods:**

We reviewed the imaging and clinical records of 48 patients (53 shoulders), 60% female, with a mean age of 63.2 years (95% CI ± 10.4 years); treated for AFTRCT who also underwent a true anteroposterior radiograph of the shoulder within a year of diagnosis of the tear. We recorded demographic (age, sex, type of work), clinical (comorbidities), and imaging data (CSA, size and location of the tear). We divided the patients into two groups according to success or failure of conservative treatment (indication for surgery), so 21 shoulders (39.6%) required surgery and were classified as failure of conservative treatment.

Univariate and multivariate analysis was performed to detect predictors of failure of conservative treatment.

**Results:**

The median CSA was 35.5º with no differences between those with failure (median 35.5º, range 29º to 48.2º) and success of conservative treatment (median 35.45º, range 30.2º to 40.3º), *p* = 0.978.

The multivariate analysis showed a younger age in patients with failure of conservative treatment (56.14 ± 9.2 vs 67.8 ± 8.4, *p* < 0.001) and that male gender was also associated with failure of conservative treatment (57% of men required surgery vs 28% of women, *p* = 0.035).

**Conclusions:**

It is still unclear if CSA does predict failure of conservative treatment. A lower age and male gender both could predicted failure of conservative treatment in AFTRCT. Further research is needed to better address this subject.

## Background

Atraumatic full thickness rotator cuff tears (AFTRCT) are frequent lesions whose incidence increases with age affecting more than 10% of patients over 60 years old [1]. Nevertheless, there is no consensus regarding the best treatment choice for these patients, and there are some debates about absolute indications of surgery [2].

Previous literature has shown that physical therapy is an effective conservative treatment in this group of patients [3, 4] with a success rate near 85% within 12 weeks of treatment (failure defined as patients choosing surgical treatment), with several factors affecting outcomes [5]. In an effort to identify predictors of failure in the conservative treatment of AFTRCT, Dunn et al. [6]. in a Neer award study showed that the main predictors were activity level, smoking status, and low patient expectations about physical therapy, while structural factors like tear size or retraction were not.

AFTRCT are a multifactorial pathology, and the influence of acromial geometry was described by Neer [7] in terms of subacromial impingement. Nyffeler [8]described an association between AFTRCT and a large lateral acromial extension, postulating that a more vertical deltoid force pulling the humeral head upward requires a larger supraspinatus counterforce to stabilize the center of rotation during abduction [9]. This concept was revisited by Moor et al. [10] who combined the lateral extension of the acromion and the upward inclination of the glenoid in a single measurement named the critical shoulder angle (CSA).

**Fig. 1:**
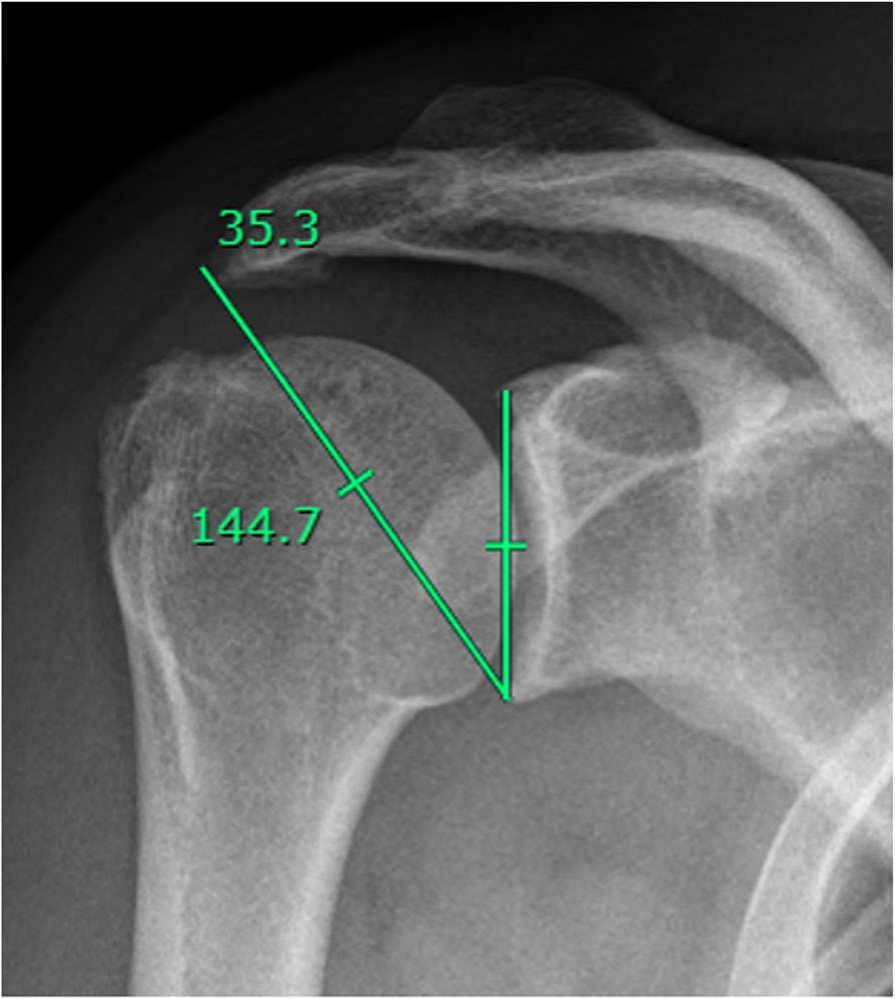
Measure of the Critical Shoulder Angle in a true anteroposterior (AP) view of the shoulder, between a line connecting the superior and inferior margins of the glenoid and another line connecting the inferior margin of the glenoid with the inferolateral aspect of the acromion

The CSA is a simple and reproducible radiographic measure made between a line connecting the superior and inferior margins of the glenoid, and another line connecting the inferior margin of the glenoid with the inferolateral aspect of the acromion (Fig. 1) in a true anteroposterior (AP) view of the shoulder.

Gerber et al. [11] and Viehöfer et al. [12] demonstrated that a greater CSA increased shear forces at the glenohumeral joint, ant suggested that this could produce a mechanical overload of the supraspinatus tendon in order to stabilize the glenohumeral joint during arm elevation.

In clinical context, some studies have shown an association between CSA and incidence of AFTRCT [13–17], and others have found an association of a large CSA and a higher risk of tendon retear [18–20].

With this previous work as backround, it might raise the question wether a larger CSA could impact clinical outcomes in the treatment of AFTRCT, more specifically, if it could predict success or failure of conservative treatment.

However, to the best of our knowledge, no study has yet described an association between a larger CSA and failure of conservatory treatment in AFTRCT.

Therefore, the main objective of this study is to determine whether there is an association between CSA and failure of physical therapy in patients with AFTRCT.

## Methods

### Patient selection and study design

Our study was performed following the STROBE statement for cohort studies and the Declaration of Helsinki. Our ethical committee approved the patient registry. This was an observational, retrospective, consecutive, monocentric, continuous multi-operator study. We reviewed data from 356 adult patients consecutively treated for an isolated full thickness posterosuperior tear diagnosed by magnetic resonance (MR) or ultrasound (US) who also underwent a true anteroposterior (AP) radiograph of the shoulder less than a year since diagnosis in a tertiary care university hospital and its primary care network from 2017 to 2018.

The patients underwent more than one image study during the review period, and only their first was used for the evaluation. The definition of AFTRCT included tears of the supraspinatus tendon extending or not to the infraspinatus tendon. Medical records were analyzed for presence of shoulder symptoms, history of trauma, comorbidities, tobacco use, physical therapy achieved, and indication of surgery within one year of the diagnostic image (definition of failure of physical therapy in patients with AFTRCT).

We excluded patients with asymptomatic tears, clear indication for surgery (eg traumatic tears), prior shoulder surgery, other causes of shoulder pain like infection, degenerative osteoarthritis, capsulitis, instability, and calcific tendonitis, and tumors. We also excluded patients that could not correctly adhere to the standardized physical therapy protocol. A total of 303 patients were excluded (Table 1) leaving 53 shoulders for statistical analysis.

**Table 1:** Excluded patients

	N	%
Clear indication for surgery (eg traumatic injury)	87	28
Prior shoulder surgery	29	10
Infection	4	1
Degenerative osteoarthritis	29	10
Capsulitis	23	8
Instability	12	4
Calcific tendonitis	27	9
Tumors	9	3
Lack of adherence to conservative treatment	83	27
Total	303	100

### Demographic data

Fifty-three shoulders of 48 patients were included: 28 were left shoulders (52.8%) and 32 patients were female (60.4%). Their mean age at the time of surgery was 63.2 years (range, 30–88 years). The demographic factors are listed in Table 2.

**Table 2:** Demographic data and distribution of patients stratified by failure of conservative treatment

Demographic data				
	Combined	Failure of conservative treatment		
		No	Yes	*p value*
Total of patients	53	32	21	
Age (mean, SD), years	63.2 ± 10.4	67.8 ± 8.4	56.14 ± 9.2	< 0.001
Gender				
Male	40% (21/53)	43% (9/21)	57% (12/21)	
Female	60% (32/53)	72% (23/32)	28% (9/32)	0.035
Shoulder involved				
Left	47% (25/53)	60% (15/25)	40% (20/25)	
Right	53% (28/53)	61% (17/28)	39% (11/28)	0.958
Manual Worker				
No	92% (46/50)	65% (30/46)	35% (16/46)	
Yes	8% (4/50)	25% (1/4)	75% (3/4)	0.147
Location of tendon tear				
Only supraspinatus	53% (28/53)	50% (14/28)	50% (14/28)	
Supraspinatus + Infraspinatus	47% (25/53)	72% (18/25)	28% (7/25)	0.102
Diabetes				
No	87% (46/53)	59% (27/46)	41% (19/46)	
Yes	13% (7/53)	71% (5/7)	29% (2/7)	0.69
Hypothyroidism				
No	84% (45/53)	58% (26/45)	42% (19/45)	
Yes	15% (8/53)	75% (6/8)	25% (2/8)	0.45
Tobacco use				
No	88% (46/52)	63% (29/46)	37% (17/46)	
Yes	12% (6/52)	50% (3/6)	50% (3/6)	0.66

### Radiographical measurements

The CSA was calculated using the technique described by Moor et al. [10] The angle was formed by a line connecting the superior and inferior bony margins of the glenoid and a line drawn from the inferior bony margin of the glenoid to the most lateral border of the acromion using the true AP shoulder radiograph (glenohumeral joint should be open; anterior and posterior aspects of the glenoid are superimposed; coracoid process is foreshortened; and no foreshortening of the scapular body). We used a cutoff of 0.25 between the transverse and longitudinal diameter of the glenoid to quantitively define a true AP view as described by Hou et al. [21].

Five evaluators (three orthopedic surgery residents, one shoulder and elbow surgery fellow, and one shoulder and elbow surgeon with 10 years of experience) independently measured each patient CSA; the mean of the four measures was used as the final result. All radiographs were obtained using a digital imaging system. The images were viewed using the Impax Web3000 program (Agfa-Gevaert, Mortsel, Belgium), which is routinely used at our institution with backup from a radiologist.

### Statistical analysis

The results are presented according to the type of variable and distribution: Continuous nonparametric data were described as median values and range, continuous parametric data were described as mean and standard deviation, and categorical variables were expressed as percentages. A univariate analysis was performed to find statistically significant differences (*p-value* less than 0.05) according to the dependent variable "Failure of conservative treatment" was defined as “indication of surgery within one year of the diagnostic image”. In this analysis, normal distribution was verified with the Shapiro–Wilk test. In those variables with normal distribution, an unpaired t-test with Welch's correction was used. The non-parametric Mann–Whitney U test was used in variables without a normal distribution. Fisher's exact test was used to compare contingency tables.

In addition, three multivariate analysis was performed for the binary dependent variable "failure of conservative treatment” using logistic regression (LR) to adjust for possible covariances: LR1 included the independent variables "CSA", "Age", "Gender", and "Tobacco use"; LR2 included the independent variables "CSA", "Age", "Gender", and "Manual worker"; and LR3 included the independent variables "CSA", "Age", "Gender", and "Location of tendon tear". Statistical analysis was performed using STATA software (version BE 17; StataCorp, College Station, TX, USA).

## Results

Of the 53 shoulders, 21 required surgery within one year of the diagnostic image and therefore were classified as failure of conservative treatment (39.6%). We measured a median CSA of 35.5 in the 53 shoulders, and there were no differences between patients who failed conservative treatment (median 35.5, range 29 to 48.2) and those who succeeded (median 35.45, range 30.2 to 40.3), *p* = 0.978 (post-hoc statistical power between 88.8 – 69.7%).

In relation to demographic and general parameters, we found that age was lower in patients who failed conservative treatment (56.14 ± 9.2, vs 67.8 ± 8.4, *p*-value < 0.001); that genre was also associated with failure of conservative treatment (57% of male patients required surgery, vs 28% of female patients, *p* = 0.035). Shoulder involvement, extension to the tear to the infraspinatus, manual working, tobacco use, diabetes, and hypothyroidism were not associated with failure of conservative treatment (Table 2). Multivariate analysis confirmed the association between age and gender in all settings. CSA was not associated with changes in failure of conservative management in any analysis.

## Discussion

In our study, we could not find an association between the magnitude of the CSA and the failure of conservative treatment in atraumatic full thickness rotator cuff tears, but instead we found that a lower age and male sex both could predict failure of conservative treatment. Our results differ from those presented by Dunn et al. [6] who found an association between tobacco use and failure of conservative treatment in AFTRCT, but failed to demonstrate an association between age and sex.

AFTRCT are lesions frequently seen in people over 60 years old, but it is still unclear what the best treatment option is for such injuries. However, more than 85% of these patients will have improved symptoms with physical therapy.

Acromial morphology has long been associated with rotator cuff disease. Initially, impingement syndrome was described as a causative factor to bursal side rotator cuff tear as proposed by Bigliani and Neer [22, 23]. This hypothesis postulated that the anterolateral narrowing of the supraspinatus outlet due to changes in acromial morphology creates an abrading effect on the rotator cuff leading to a subsequent tear. However, several authors concluded that acquired changes within the acromion and coracoacromial ligament impact rotator cuff disorders rather than cause the disorders [24–27]. Finally, randomized clinical trials [28] and a meta-analysis [29] demonstrated no difference in outcome and failure rate between repairs regardless of whether an anterolateral acromioplasty was performed.

Nyffeler et al. [8] proposed a theory based on the lateral extension of the acromion and the resulting vertical force of the acromion during abduction; increased strain or shear force was needed to stabilize the humeral head by the supraspinatus. The authors found an association between a large lateral extension of the acromion and symptomatic rotator cuff tears. A superior glenoid orientation also accounts for this phenomena, and its association with rotator cuff tears has also been demonstrated [30–32].

Moor et al. [10] developed the CSA to consider both components in a single angle. In their milestone paper, this group reported a larger CSA (38º vs 31º) in patients with degenerative rotator cuff tears compared with controls and postulated that CSA may be a risk factor for developing degenerative rotator cuff tears. Several authors have accounted for this relationship [13, 33–37].

Biomechanical models have demonstrated the mechanistic basis of the pathology of rotator cuff tears underlying the scapular anatomical differences. Gerber et al. [11] used a simplified robotic model to document that a greater CSA increased shear forces (decreased compressive forces) at the glenohumeral joint requiring an 35% additional force of the supraspinatus to achieve stability at relatively low angles of thoraco-humeral abduction (40º). In a 3D Finite element model, Viehöfer et al. [12] demonstrated that a higher CSA has a substantial effect on the middle deltoid wrapping and force of action leading to a higher shear and smaller joint compression force during active abduction. Those studies suggested that a smaller CSA requires a lower rotator cuff force to provide glenohumeral stability during active elevation.

Furthermore, it is possible that an individual with a rotator cuff tear and a smaller CSA would be more prone to compensate this instability with other co-contracting muscles and have a favorable outcome; in other scenarios, a patient with a higher CSA would be more difficult to compensate and stabilize the shear forces of the deltoid with the other co-contracting muscles. However, this hypothesis has not been confirmed here.

### Our study has several limitations

First, it is a retrospective study of patients at a single tertiary care university hospital and its network. Due to our strict inclusion criteria, we had to exclude most patients initially selected. Moreover, we studied only patients with posterosuperior tears (tear of the supraspinatus extending or not to the infraspinatus). Patients with subscapularis tears were excluded. As a result, we only included 53 patients in our final analysis, and this could lead to selection bias in favor of patients with failure of conservative treatment, and this could explain why we observed a smaller proportion of patient who responded to conservative treatment compared to previous studies.

Second, our sample size was small, and the post hoc analysis we observed a power of only 20%.

Third, it is known that the treatment decision in AFTRCT is multifactorial and involves factors such as the patient's lifestyle, expectations, degree of pain and dysfunction, clinical findings (eg range of motion, pseudoparalysis, weakness) and structural findings tear (size, retraction, degree of glenohumeral osteoarthritis).

Taking this into account, trying to predict the failure of conservative treatment in this pathology using a single structural factor such as CSA could be simplistic. For this reason, also taking into account our low sample size the low quality of evidence provided by a retrospective study, we believe that studies with higher quality of evidence and involving more factors are required to more holistically determine the factors associated with failure of conservative treatment in AFTRCT. We hope that our work can be used as a basis for future studies on this subject, as maybe with the same method and more sample size a better conclusion could be made. We also summarized previous literature on the subject.

## Conclusions

It is still unclear if CSA does predict failure of conservative treatment. A lower age and male gender both could predicted failure of conservative treatment in AFTRCT. Further research is needed to better address this subject.

## Data Availability

The datasets used and/or analyzed during the current study are available from the corresponding author on reasonable request.

## Abbreviations

AFTRCT: Atraumatic full thickness rotator cuff tears
MR: Magnetic resonance
US: Ultrasound
AP: Anteroposterior
CSA: Critical shoulder angle
